# Leishmaniasis Beyond East Africa

**DOI:** 10.3389/fvets.2021.618766

**Published:** 2021-02-26

**Authors:** Caitlin M. Jones, Susan C. Welburn

**Affiliations:** ^1^Zhejiang University-University of Edinburgh Institute, Zhejiang University School of Medicine, International Campus, Zhejiang University, Haining, China; ^2^Infection Medicine, Deanery of Biomedical Sciences, Edinburgh Medical School, College of Medicine and Veterinary Medicine, The University of Edinburgh, Edinburgh, United Kingdom

**Keywords:** leishmaniasis, climate, epidemiology, Africa, visceral, cutaneous, vector

## Abstract

Climate change is having a substantial impact on our environment and ecosystems and has altered the way humans live, access, and utilize resources with increased risk of zoonotic infectious disease encounters. As global temperatures continue to increase, they impact on public health, migration, food security and land conflict, and as new environments become favorable, exposure to disease carrying vectors. Increased forests or natural habitat clearance for land repurposing, urbanization, road building, and water management are related to an increase in emerging vector borne parasitic diseases. The East African region remains one of the most impacted regions globally for leishmaniasis, a vector borne disease that impacts significantly on the health, wellbeing and livelihoods of affected communities and for which a lack of reporting and control interventions hinder progress toward elimination of this neglected tropical disease. As our world continues to transform, both politically and climatically, it is essential that measures are put in place to improve surveillance and disease management with implementation of control measures, including vector control, especially in low- and middle-income countries that are expected to be most impacted by changes in climate. Only through effective management, now, can we be sufficiently resilient to preventing the inevitable spread of vectors into suitable habitat and expansion of the geographical range of leishmaniasis. This review offers a current perspective on Leishmaniasis as an endemic disease in East Africa and examines the potential of the recent emergence of *Leishmania* infection in hitherto unaffected regions to become a public health concern if no disease management is achieved.

## Background

Each year, neglected tropical diseases have a substantial impact on upwards of 1 billion people globally, contributing to the damage of economies and impairment of particularly vulnerable communities including those living in extreme poverty (1). The leishmaniases are estimated to affect 0.7–1 million people, with 350 million people at risk, globally (2, 3). Currently, 98 countries are endemic for leishmaniasis (2). Leishmaniasis occurs after the infection of a mammalian host with the obligate, intracellular parasite *Leishmania* (4). *Leishmania* species are categorized into either Old World or New World species, corresponding to the different geographical regions in which they are found (5). Old World (OW) species can be found in Asia, the Middle East, the Mediterranean basin and Africa, whereas New World (NW) species are found in the Americas (5). At least 20 *Leishmania* species are known to cause human disease, of the *Leishmania* genus and *Leishmania* or *Viannia* subgenera (6, 7). The subgenera were distinguished in the 1980's based on parasite location and site of attachment within the sand-fly gut (7).

*Leishmania* parasites can infect over 90 sandfly species; the *Phlebotomus* and *Lutzomyia* species being most implicated in human leishmaniasis transmission (8). The manifestation of leishmanial disease can occur when parasites are transmitted to the mammalian host upon the infected female sandfly taking a blood meal, which provides the protein required for egg development (9). The dimorphic parasites are transmitted to the human host in their motile, metacyclic promastigote form, invade phagocytic cells—predominantly macrophages—and transform into their non-motile amastigote form (4). Within the cell the amastigotes replicate by binary fission until cell rupture and proceed to further invade healthy neighboring phagocytes (4).

Disease outcome is dependent on a number factors, including parasite species, host genetics and the host immune responses (10). Disease severity can range from cases being serologically positive yet asymptomatic, to cases of disfiguring and fatal infection (11). The three disease forms are characterized as cutaneous, mucocutaneous and visceral leishmaniasis (2). Several complications are also associated, such as post-kala-azar dermal leishmaniasis (PKDL), diffuse leishmaniasis, disseminated leishmaniasis and leishmaniasis recidivans (12). Cutaneous leishmaniasis (CL) is considered the mildest and most common form of the disease, and can present as painless nodules, macules, papules or ulcerative lesions on exposed areas of the body (13, 14).

There are more than 20 species that cause cutaneous leishmaniasis, most notably the OW species: *L. major, L. tropica, L. aethiopica*, and the NW species: *L. amazonesis, L. braziliensis, L. mexicana, L. panamensis*, and *L. guyanensis* (2, 15). Lesions are local to the site of the sandfly bite and can be self-healing, however the appearance and healing time varies between species (16). While cutaneous lesions due to *L. tropica* or *L. major* are likely to self-heal after 12 months, leaving significant scarring, *L. aethopica* lesions have been shown to become plaque-like and hyperkeratotic, taking many years to heal completely (17, 18). Although not fatal, cutaneous leishmaniasis can lead to serious scarring or disfigurement, which can result in significant psychological stress, including depression and anxiety and social stigmatization leading to a poor quality of life (19–21). Presently, there are 59 countries where the cutaneous form is endemic, 10 of which (Afghanistan, Algeria, Bolivia, Brazil, Colombia, Iran, Iraq, Pakistan, the Syrian Arab Republic and Tunisia) are responsible for 85% of all recorded cases (22).

Mucocutaneous leishmaniasis (MCL) involves the destruction of mucus membranes and cartilage in the nose, mouth, upper respiratory tract and pharynx, often beginning at the lips and nostrils (23). Although the pathology of MCL is poorly understood, it is thought to be, in part, due to a strong host immunological response (23, 24). It has been suggested that parasites may reach mucosa either by haematic or lymphatic spread or are directly injected into the area by the sandfly (23). Symptoms of MCL can arise years after an initial cutaneous lesion has healed, include discharge, congestion, and acute hemorrhage of the nose (25, 26). This can be extremely disfiguring as well as life-threatening, potentially leading to complications such as aspiration pneumonia, starvation, sepsis due to secondary infection, and airway obstruction thereby resulting in asphyxia (27). Of all mucocutaneous cases, 90% are found in Bolivia caused by NW species, however *L. aethiopica* has also demonstrated the ability to cause MCL in Ethiopia, a country in the OW Region (2, 27).

Visceral leishmaniasis (VL) occurs after the systemic spread of *Leishmania* parasites and can be life-threatening (28, 29). VL is characterized by presence of an enlarged liver and spleen, anemia, weight loss, and irregular fevers (2). Patients with VL often die within 2 years of contracting the disease if left untreated, commonly from subsequent infections or severe anemia (30, 31). Although not seen in all VL species it is expected that darkening of the skin, particularly in India, is caused by the cytokine-induced production of adrenocorticotrophic hormone (32). Thus, VL was given the Hindu name kala-azar, meaning “black fever” (33). Two species are known to cause human VL, where *L. donovani* predominantly affects the OW and *L. infantum* (synonym *L. chagasi)* the NW regions (34).

There are 59 countries endemic for VL, although cases fall disproportionately on seven of these, with 90% of all cases found in Brazil, Ethiopia, India, Kenya, Somalia, South Sudan and Sudan (22). There are an estimated 20,000–40,000 deaths annually due to VL, however this figure is likely to be higher as it is likely that the vast majority of VL deaths are not recognized as being caused by leishmaniasis or are not reported (35). Leishmaniasis is well-documented in several countries, including India, which carries one of the highest burdens of leishmaniasis globally, followed by Sudan (36). In other areas of the world, the epidemiology of this disease remains poorly understood.

## Leishmaniasis, An Endemic Public Health Problem in East Africa

In the East African region, cutaneous and visceral leishmaniasis have numerous strongholds across Ethiopia, Sudan, Uganda and Kenya, Somalia, and Eritrea (37).

## Ethiopia

Ethiopia is a landlocked country found in the Horn of Africa peninsula (38), endemic for both CL and VL (39). Leishmaniasis has been long established in Ethiopia, with the first scientific reports of CL dating back to 1913 and VL in 1942 (40, 41). It is estimated that Ethiopia accounts for surplus of 30,000–50,000 cases of CL and 3,700–7,400 cases of VL annually (21, 42). In the East African region, Ethiopia has the second largest number of VL cases a year, behind Sudan (43). The incidence rate per 10,000 people in endemic areas is 6.28 for VL and 1.05 for CL (44). Nationally, over 28–30 million people are considered to live in areas that put them at risk of contracting leishmaniasis (45, 46). The major causative species in this region are *L. donovani* for VL (though *L. infantum* has been recorded) and *L. aethiopica* for CL (*L. major* and *L. tropica* have also less frequently been reported) (35, 47, 48). An official country-wide or state level prevalence for either form is not reported, however a recent systematic review reported a national pooled prevalence of 19% (95% CI = 14–25%) (49).

Currently, there is no active case screening for leishmaniasis, only tracking of active cases, and so the basal pool of cases may be much greater than expected (38, 44). VL has been identified in eight states: the northern states of Tigray, Amhara, Afar, Benshangul-Gumuz and the southern states of Oromia, Gambella, Somali, and the Southern Nations and Nationalities People's Regional State (SNNPR) (49). There are several well-established endemic VL foci, including the Metema and Humera plains in the NW of the country which accounts for around 60% of cases, as well as the Omo plains and the Weyto Valley in the south (50–52). Approximately 20% of VL cases occur in the south western savannah and semi-arid lowlands in the SE (53). VL cases have also been recorded in the surrounding areas of Moyale and the Genale river basins in the south of Oromia, in the Afder and Liben zones in the south eastern Somali state and the Awash Valley in the northern state of Afar (43, 49, 54). Despite VL being predominantly found in the lowlands, there have been outbreaks recorded in the previous unaffected highland area of Libo Kemkem (altitude above 2,000 m) in 2005 and Belessa in 1970, both in Amhara (54, 55). In contrast to VL, the cutaneous form of disease is strongly associated with the highland regions of Ethiopia where altitudes are above 1,400 m (21, 51). It is expected that CL is present nationally, however well-recognized foci include Ochollo of the Rift Valley, Kutaber district in Amhara, Aleku, Sebeta, the Bale and Sidamo highlands of Oromia, and the Adi-grat and Saesie Tsaedaemba districts of Tigray (39, 56–59).

Transmission of *Leishmania* species that have the potential to cause VL or CL can be zoonotic or anthroponotic [49]. Transmission is not fully understood in Ethiopia, although *L. aethiopica*, which can cause CL, is thought to primarily be zoonotic [49, 56]. A reservoir for the CL causative agent *L. aethiopica* has been identified as the rock hyrax, with two species primarily involved: *Heterohyrax brucei* and *Procavia capensis* (60, 61). Transmission of VL caused by *L. donovani* is generally considered to be anthroponotic in the East African region, however, in Ethiopia it is thought to be partially zoonotic and partially anthroponotic, the nature of which varies with geographical areas (62). Animal reservoirs of *Leishmania* species causing VL have not been definitively identified, although dogs are likely candidates, as is the case in the neighboring country of Sudan (54, 63). Other potential reservoirs are squirrels, wild canids, rodents, reptiles and bats (39, 53, 64–66).

## Sudan

To the west of Ethiopia, Sudan is highly endemic for both CL and VL. Although initial reports of VL date back to 1904, it is thought that the *Leishmania* parasite has been present in Sudan for over 4,000 years, with parasite DNA having been detected in the bone marrow of mummies from this time (67, 68). Annually, Sudan accounts for roughly two thirds of all reported VL cases in East Africa (65). Since 2015, around 93% of the population of Sudan is considered at risk of CL and 25% at risk of VL (69), despite having incidence rates that are lower than that for Ethiopia in 2015 (3.25 per 10,000 for VL and 0.94 per 10,000 for CL) likely due to significant underreporting at all levels as a function of poor infrastructure (38) and the lack of ability to diagnose cases locally (38, 65, 69, 70). Additionally, this is mirrored in the number of new cases reported in 2015, with only 2,829 cases of VL and 3,503 of CL having been reported by the WHO (69). There is currently no active screening for the control of leishmaniasis in Sudan, but passive case detection and subsequent treatment (71).

The parasite species responsible for VL in Sudan is predominantly *L. donovani*, however, sporadic detection of *L. archibaldi* and *L. infantum* has occurred in humans and dogs in the State of Gedaref (72). In Sudan, *L. donovani* is transmitted to the human by the sandfly species *P. orientalis*, though the presence of *P. martini* has also been reported (72). Regions endemic for VL are found in the north and east of Sudan, particularly along the border with northern Ethiopia, including the Sudanese states of Gedaref, Senna, Al Qadarif, and Blue Nile (73). Villages close to the Ethiopian border, concentrated along the Atbarah and Rahad rivers are particularly affected by VL (73). In contrast, CL which is caused by *L. major* in this region, can be found in the central and western states of Sudan, including Northern and Southern Dafur, Northern and Southern Kordofan, Khartoum, and El Gezira (74). In Sudan, *L. major* is transmitted by the sandfly species *P. papatasi* (75, 76). A recent study utilizing surveillance data reported that the number of CL cases reported from these states in 2014–2017 increased annually, greatly exceeding the estimates reported by the WHO in 2014 (74). Whilst the aforementioned species causing visceral and cutaneous leishmaniasis in Sudan are considered anthroponotic, mammals including the Egyptian mongoose and dogs have also been investigated as parasite reservoir hosts (63, 77, 78).

## Kenya

VL is endemic in the arid and semi-arid regions of Kenya including the Rift Valley and provinces in the east and north east of Kenya caused by *L. donovani* (79, 80). Confirmed VL endemic regions are focussed in the arid lowlands, and include Baringo, Turkana, Marakwet, Smaburu, Pokot, Laikipia and Kajiado, Machakos, Mwingi, Meru, Wajir, and Keiyo (79–81). Baringo County is a well-established focus for both CL and VL (82). The West Pokot focus stretches across the border into the Nakapiripirit district of Karamoja region, Uganda, where the most Ugandan cases are concentrated (83). Since its first report in 1935, there have been several epidemics of VL in Kenya, including in the previously unaffected areas of Wajir and Mandera during 2000–2001 (80). It is expected that there are around 4,000 cases of VL annually (84). Over 5 million people in Kenya are considered at risk of exposure to leishmaniasis, with an incidence rate of 2.96 per 10,000 people for VL, however, incidence rate for CL is not reported (84, 85).

In Kenya, the cutaneous form of the disease is deemed endemic by the WHO, however there are a lack of accurate data describing the true extent of disease or case numbers (85, 86). CL was first documented in 1969 and is caused by: *L. tropica*, predominantly found centrally and in the Rift Valley; *L. major*, reported in the lowlands of Kitui and Baringo (38, 82, 87); and *L. aethiopica*, detected in areas of high altitudes such as Mount Elgon (88). The vectors responsible for transmission in these regions are *Phlebotomus duboscqi, Phlebotomus guggisbergi*, and *Phlebotomus pedifer* (89). In some instances, there have been cases where individuals have been positive for both *L. major* and *L. tropica* (80). Transmission of *L. aethiopica, L. major* or *L. tropica* in Kenya is primarily thought to be zoonotic, with rodents, rock hyrax and dog being implicated in the transmission of parasites to humans (82, 90, 91).

## Uganda

In Uganda, the visceral form of disease is endemic and was first reported in 1951 in the north east region of Karamoja (92). Familiarity of VL within the community is evident as it is known vernacularly as “Termes” to the Pokot people of Uganda (93). Several foci of VL are located across the Amarut, Moroto, Kotido, and Nakapiripirit districts of the Karamoja region which lies on the Kenyan border, where residents freely move between Uganda and the West Pokot and Baringo counties of Kenya (93, 94). It is thought that the vector involved in VL transmission to humans in this region is *P. martini*, similarly to Kenya, and the infective parasite species is *L. donovani*. There is currently a lack of data describing any possible reservoir hosts for *L. donovani*, as well as prevalence, incidence rates or risk factors in these endemic areas (93).

## Somalia

The WHO reports that VL, but not CL, is endemic in Somalia, where incidence is 4.98 per 10,000 of the population in endemic areas (95). The causative species of VL in Somalia is identified as the *L. donovani* complex (96). Despite no definitive data confirming the competent vector, it is suspected that *P. martini* is a likely candidate in endemic areas due to few studies identifying its presence (97). As with most East African countries, VL is likely to be anthroponotic in Somalia, with no definite animal reservoir reported. Somalia has suffered from conflict for many years, which has restricted access to health care and impeded efforts for control and surveillance of infectious diseases, with the only current conceivable method of control being active case treatment (98). Healthcare provided by non-governmental organizations such as Médecins Sans Frontières (MSF), was withdrawn in 2013 due to ongoing violence and attacks on MSF workers rendering up to 1.5 million locals without health care and vulnerable to infectious diseases (99). The availability of epidemiological data for leishmaniasis in Somalia is rare (98). A few reports have documented VL endemic areas in Baidoa, lower Juba and middle Shebelle river, one citing the prevalence of VL seropositivity as high as 23% in one village (98, 100, 101).

## Eritrea

In Eritrea, there is a significant lack of data on the epidemiology of leishmaniasis. Few cases have been reported, mainly originating from the administrative regions on the Ethiopian and Sudanese side of the border, with a more recent case being described in the capital, Asmara (38, 102). Upon detection of leishmaniasis in Eritrea, the disease form is not distinguished and most commonly recorded as VL (103). Therefore, the overall extent of both disease forms is not clear (103). Currently, there are no control programmes, data defining vector species or reservoir hosts for *Leishmania* parasites in Eritrea (103).

## Risk Factors of Disease

Children and young adults are demographically most at risk of developing symptomatic leishmaniasis in endemic areas, potentially due to lack of protective immunity or due to them collecting water or playing in gorges close to sandfly habitat (48, 54, 60). In areas with active outbreaks, all ages are at risk, with risk increasing with exposure to the vector (104).

Males may be more exposed than females, since they are more involved in outdoor agriculture or activity (38). Agriculture workers commonly work at night which coincides with the peak vector activity (53). Seasonal agricultural work sees a large influx of people moving from unaffected areas to endemic areas, such as the Ethiopian Humera plains near the Sudanese border, putting individuals at risk of VL (105). Similarly, infected individuals migrating from VL hotspots back to their homes serve as reservoirs for establishing new disease foci in previously unaffected areas (106).

Incidence in the north of Ethiopia is associated with cracking black cotton soil and Acacia-Balanite trees and termite hills and reddish clay soil in the south—the variation being due to the breeding site preferences of varying sandfly species (64).

Civil unrest, such as that seen in Sudan, can result in forced migration from endemic areas to non-endemic, further spreading the disease (107, 108). From 1983 until 2005, civil war between Sudan and South Sudan resulted in the uprooting and displacement of over 4 million people, which coincided with VL outbreaks and consequently 100,000 deaths over the 22 year period (65, 109, 110). In subsequent years tension and unrest persisted, forcing millions of people to flee, with many immunologically naïve individuals migrating to endemic regions of neighboring countries such as the Omo plains or the Rift Valley (65). From the resumption of unrest in 2013, MSF reported that VL cases doubled in three endemic areas by 2014 (65).

Living near to, resting by or sleeping under Acacia-Balanite trees will increase the likelihood of exposure to the vector and thereby increase risk of disease in Ethiopia, Kenya, Somalia, and the Sudanese-Ethiopian borders (105). Additionally, proximity to dogs, cattle or termite hills increase the risk of exposure in endemic areas such as Libo Kemkem in Ethiopia (105).

Some species of sandfly are expected to be exophagic, and so sleeping outside increases the chance of sandfly bite (42, 111). Those subjected to living in poverty often own poor-quality housing (20). Houses with thatch walls or with no protective measures in place, such as bed nets or curtains, are more likely to be exposed to the vector (105, 108). With poverty often comes malnutrition, including lack of protein, iron, vitamin A and zinc levels which has been linked to VL development (54, 112). HIV-VL co-endemicity in north-west Ethiopia has resulted in coinfection rates of 20–40%, where the associated immunosuppression aids VL development and HIV progression (113, 114). Other proposed risk factors include implementation of irrigation systems, the development of the sugar industry and forest clearance (45).

## Climate Change and Leishmaniasis

Increased global temperatures have resulted in increased flooding, droughts, land fires, and other destructive natural disasters. Since the 1880s, the temperature has been steadily rising in increments of 0.07°C, up until the past four decades, where this increased to 0.18°C (115). It is expected that climate warming will continue throughout the 21st century, with higher latitudes being more heavily impacted (116). Global warming has had a substantial impact on our environment and ecosystems and has altered the way humans live and move around. These two factors combined mean that human and infectious disease encounters will become more frequent and new environments will become more favorable to disease carrying vectors. Increased forest area clearance for land repurposing, urbanization, the building of roads, and water management structures are mirrored by an increase in emerging parasitic diseases (117).

The arthropod vectors responsible for transmitting the parasite, sandflies, are greatly impacted by changes to their ecology, resulting in changes in vector numbers, species present within an area, the mix of species within an area, and vector behaviors including, feeding, resting, or activity periods (117). In the OW, there are 31 proven sandfly species with the ability to transmit *Leishmania* parasites (6). Knowledge of vector behavior is a crucial part of understanding disease transmission and allows for the development of vector control strategies (118). Sandflies are commonly found in warm countries with tropical or subtropical climates, notably Australia, the Americas, Asia, Africa, and the south of Europe, between 50°N and 40°S (119). Sandflies are considered thermophilic and so necessitate an environment where the average temperature is consistently high, as temperatures below 15°C will reduce survival rates in several species (120, 121).

Parasite development within the sandfly is optimum at temperatures around 25°C, where higher temperatures decrease incubation time and permit faster transmission through increased reproduction in the midgut and movement through the sandfly (122). Heavy precipitation brought about by increased global temperatures will decrease flight ability, availability of landing surfaces, and can be fatal to larvae, however, some moisture within the soil of the sandfly environment is still required for survival. Despite this, as global warming intensifies, soil is likely to lack the required moisture due to fast evaporation rates as a result of long periods of sustained high temperatures and drought.

## Leishmaniasis, An Emerging Public Health Problem

In the WHO European region, CL is endemic in Israel, Turkey, Uzbekistan, and Turkmenistan, whereas VL is much more widespread in the south-west of Europe, the Balkans, Turkey, Caucasus, and central Asia; with over 70% of VL cases in Italy, Spain, Albania, and Georgia (123). The parasites found in this region are *L*. *infantum, L. tropica*, and *L. major*, where CL is caused by all three species and VL by solely by *L. infantum* (123). The northern limit of sandfly distribution in Europe previously covered the south Mediterranean region and south eastern Europe (124). In recent years, there have been increases in the number of sporadic cases of leishmaniasis (cases identified by PCR as *L. infantum* or *L. donovani*/*L. infantum*) being reported from countries in central Europe including Germany, Austria, and in southern England (124–126). In southern European countries, there are several established sandfly species that are known to have the ability to host human-infective *Leishmania* parasites, including *P. sergenti, P. papatasi, P. alexandri, P. tobbi, P. perniciosus, P. ariasi, P. perfiliewi*, and *P. neglectus* (120). A rise in temperature by 1°C is suitable for survival of Phlebotomine sandflies in parts of Austria, such as the Slovenian border region, in which foci of leishmaniasis are likely to develop supported by canine reservoirs (124). Additionally, *L. infantum* DNA was detected from a caught sandfly (*P. mascittii*) in Austria (127). By 2040 more areas in the west and northwest of Germany are expected to become moderately or highly suitable for sandfly species survival (128). Sandflies were considered absent in Germany prior to 1999 but have now been found in Baden-Württemberg and were identified at 37 different sites in the Rhine Valley, Southwestern Germany (129, 130). Although the predominant species identified in this area is *P.mascittii*, in 2001 *P. perniciosus* sandflies were reported for the first time which have shown the ability to host *L. infantum* parasites (130). A recent ecological niche modeling study reported that the future climatic conditions could provide a suitable environment for sandfly species as far north as the UK and Scandinavia (*P. papatasi, P. tobbi, P. perniciosus, P.ariasi, P. perfiliewi, P. mascittii*, and *P. neglectus*) (120). This is supported by another bioclimatic envelope modelling study which found that the central European climate will become more suitable for sandfly species currently found in the south-west of Europe, however dispersal of sandflies may also be inhibited by geographical barriers such as the Alps and sandfly flight ability (131). Around 100,000 dogs in Germany are estimated to be carrying *Leishmania* parasites (*L. infantum*) which could, given that there are competent vectors, act as reservoirs across the country (130). In addition to this, studies have revealed high numbers of asymptomatic *L.infantum* carriers in endemic regions, which suggests that humans could also act as reservoirs in new European foci (132).

The World Health Assembly resolution WHA 60.13 and WHO expert committee brought attention to the necessity leishmaniasis for epidemiology research, particularly in the European region, so that efficient policies, guidelines, and control strategies could be implemented to prevent further disease dissemination (123, 133). The WHO aim to prevent further spread of leishmaniasis by introducing surveillance, encouraging research on diagnostics and therapeutics, improving communication between collaborators and having epidemic management plans in place (123). Given the changes that are expected with climate change, it is fathomable that leishmaniasis could become a public health concern not only in the south of Europe, but central and north also. Suitable conditions for sandfly survival incorporated with factors such as increase in global travel and importation of reservoirs, such as dogs, from endemic countries where the disease is not efficiently managed could contribute to leishmaniasis outbreaks in these previously non-endemic countries. Similarly, in the Americas, with leishmaniasis already detected in Texas and Ohio, it has been predicted that the number of people exposed to leishmaniasis in North America will double by 2080 as leishmaniasis moves further north into the Americas, affecting east central North America and potentially southern Canada (134, 135). Ecological niche modeling of the impact climate change may have on both suspected vectors, *Lutzomyia diabolica* and *Lutzomyia anthophora*, and the woodrat reservoirs indicated their expansion of their habitats in North America, providing a wider environment suitable for *Leishmania mexicana* parasites (134). Although generally considered an imported disease in the US, either from travelers, the military or migrants, a study conducted in Texas demonstrated that 59% of identified leishmaniasis cases were autochthonous (32% of these cases were identified as *L. mexicana*, the other cases did not provide species information) (136). The underlying circulation of *Leishmania* parasites in animal reservoirs in this area may provide a starting point for expansion across North America as climate change ensues and vector and reservoir habitats expand. Despite this, in the US there is a possibility that the sandfly expansion can also be hindered, due to factors such as unsuitable landscape, species competition, and also geographical barriers (134).

## Conclusion

Leishmaniasis remains a severe public health threat, particularly for those afflicted by poverty, war, living in conditions of poor nutrition and with impaired health systems. The East African region remains one of the most impacted regions globally and a lack of reporting with regards to the prevalence, distribution, social/risk factors, and transmission of leishmaniasis continues to be a constraint to disease management in most afflicted countries. As our world continues to transform, both politically and climatically, it is essential that we understand the current epidemiology of leishmaniasis in endemic regions, that we improve surveillance and disease management and implement vector control where appropriate, especially in the low- and middle-income countries that are expected to be most impacted by changes in climate. Sporadic cases and *Leishmania* parasites have already been identified in canines in non-endemic central European countries, as well as southern states of the US. Regions of the world previously considered unaffected by leishmaniasis are predicted to become suitable to vector and reservoir of *Leishmania* species. Without a proper understanding of the disease in the endemic setting, we lack the ability to plan and efficiently implement disease control. For the leishmaniases, as for other neglected zoonoses, intervention costs can seem high when compared to the public health benefits alone. However, these costs are easily outweighed when a full cross-sector analysis is carried out and the monetary and non-monetary benefits to all stakeholders are taken into account. Only through effective management now, can we be sufficiently resilient to prevent the inevitable spread of infection with increased suitable vector habitat and prevention of expansion of the geographical range for leishmaniasis.

## Author Contributions

CJ and SW were both involved in conception, design, and writing of this perspective. Both authors read and approved the final version of the manuscript.

## Conflict of Interest

The authors declare that the research was conducted in the absence of any commercial or financial relationships that could be construed as a potential conflict of interest.
